# Cross-cultural adaptation and validation of the Indonesian version of AQUAREL on patients with permanent pacemaker: a cross-sectional study

**DOI:** 10.1186/s13104-019-4208-1

**Published:** 2019-03-28

**Authors:** Muhammad Yamin, Simon Salim, Siti Setiati, Idrus Alwi, Putri Zulmiyusrini

**Affiliations:** 1 Division of Cardiology, Department of Internal Medicine, Faculty of Medicine Universitas Indonesia/Cipto Mangunkusumo National General Hospital, Jakarta, Indonesia; 2 Clinical Epidemiology and Evidence-based Medicine Unit, Cipto Mangunkusumo National General Hospital, Jakarta, Indonesia; 3 Department of Internal Medicine, Faculty of Medicine Universitas Indonesia/Cipto Mangunkusumo National General Hospital, Jakarta, Indonesia

**Keywords:** Quality of life, Questionnaire, AQUAREL, Pacemaker, Indonesia

## Abstract

**Objective:**

The assessment of quality of life has significant impact in device therapy. This research was aimed to translate and evaluate the validity and reliability of the Indonesian version of the AQUAREL questionnaire.

**Results:**

We evaluated 32 patients during the cross-cultural adaptation stage and 20 patients during validity and reliability evaluation stages. Indonesian version of AQUAREL showed positive correlation between 6-min walking test and dyspnea domain (r = 0.228; p = 0.048), and showed negative correlation between NT pro-BNP and chest discomfort (r = − 0.231; p = 0.043) and dyspnea domain (r = − 0.268; p = 0.020). The total AQUAREL also showed positive moderate correlation toward total SF-36 (r = 0.543; p = 0.000). The internal consistency was good (Cronbach α = 0.728) and the repeatability between day 1 and day 8 was good, with moderate positive correlation (r = 0.581; p = 0.007).

**Electronic supplementary material:**

The online version of this article (10.1186/s13104-019-4208-1) contains supplementary material, which is available to authorized users.

## Introduction

Pacemaker therapy is increasingly done worldwide [1]. Initially to add years in patient’s life, its aim is now combined to add quality in life/Quality Adjusted Life Year (QALY) [2]. Although mortality benefit of this treatment is undoubtful [3, 4], patient’s perspective will influence the benefit of improving quality of life.

Health Related Quality of life (HRQOL) can be assessed by means of generic and specific questionnaires. Short Form-36 (SF-36) as one of the generic questionnaires for HRQOL has already been successfully translated into Indonesian language [5]. There’s no specific questionnaire for pacemaker patient’s in Indonesian Language, even though it’s best to use generic and specific questionnaire together in evaluating patients [6]. Assessment of Quality of Life and Related Events (AQUAREL) (Julius Center for Health Sciences and Primary Care, Utrecht University and Heart Lung Center Utrecht, The Netherlands) was developed specifically for pacemaker patients [7, 8]. Thus, we aim to adapt AQUAREL into Bahasa Indonesia and evaluate its validity and reliability.

## Main text

### Methods

A cross-sectional study was performed between September 2014–August 2016 in outpatient cardiology clinic, Cipto Mangunkusumo National General Hospital, Jakarta, Indonesia. Patients aged > 18 years with > 3 months pacemaker implantation were enrolled. Patients with congestive heart failure NYHA III–IV, cognitive impairment, physical disability, non-optimal echo window, and other comorbidities such as pericardial and pleural effusion were excluded. Our translation and cultural adaptation of AQUAREL into Indonesian language follow the method outlined by Guillemin and Beaton [9, 10] which have been reviewed by others [11, 12] and has been described in detail in our group previous publication [5].

In short, it was divided into 2 phases. Phase 1 (Translation and Cross-Cultural) consisted of 6 stages: initial translation, translation synthesis, back translation, committee review, pretesting, and submission and appraisal of written reports to committee. The initial translation was performed by native Indonesian-speaker (Additional file 1: Appendixes S19, S20) and back translation completed by native English-speaker translators (Additional file 1: Appendixes S22, S23).

Phase 2 (Validity and Reliability) consisted of two procedures. The validity test was done by comparing AQUAREL against Indonesian SF-36, 6 min-walking test (6MWT) and NT-ProBNP with Kendall Tau’s correlation. The reliability of the Indonesian version of AQUAREL (Indonesian-AQUAREL) was assessed using Cronbach-α for internal consistency, Kendall’s Tau, Wilcoxon Signed Rank and intraclass correlation coefficient (ICC) were used for test–retest reliability comparing results of day 1 and 8.

Sample size for phase 1 was estimated to 32 patients [13] and 20 patients for phase 2 [14]. The statistical analyses were conducted using SPSS statistics software v.23.0.

### Results

There were 32 patients participated in translation-cultural adaptation process and 20 patients included in validity-reliability testing of Indonesian version of AQUAREL. The characteristics of the patients can be seen in Table 1.

**Table 1:** Patients’ characteristics in language–cultural adaptation and validity–reliability test of Indonesian version of AQUAREL

Characteristics	Language and cultural adaptation n = 32	Validity and reliability test n = 20
Age (mean ± SD)	65.8 ± 15.096	62.35 ± 16.69
Gender (%)		
Male	46.9	55.0
Female	53.1	45.0
Educational background (%)		
Elementary school	18.8	25.0
Junior high school	21.9	30.0
Senior high school	21.9	10.0
Diploma	9.3	5.0
Bachelor degree	28.1	25.0
Master degree	0	5.0
Indication for pacemaker (%)		
AV block (high degree or total)	62.5	75.0
Sick sinus syndrome	25.0	10.0
Symptomatic bradycardia	12.5	15.0
Type of permanent pacemaker (%)		15.0
Single chamber	18.7	0
Double chamber	81.3	100.0

AQUAREL consisted of 20 items within 3 domains: chest discomfort or CHS (8 items), dyspnea and exertion or DYS (7 items), and arrhythmia or ARR (5 items).

#### Correlation between Indonesian Version of AQUAREL and SF-36

SF-36 consists of 36 items, which are classified in 8 domains: general health (GH), physical functioning (PF), role-physical (RP), bodily pain (BP), vitality (VT), social functioning (SF), role-emotional (RE), and mental health (MH) [8]. Correlation between 3 domains AQUAREL and 8 domains SF-36 was assessed by Kendall’s Tau non-parametric test (Additional file 1: Appendix S7). Correlation was categorized very strong (r = 0.80–1.00); strong (r = 0.60–0.79); moderate (r = 0.40–0.59); weak (r = 0.20–0.39); and very weak (r = 0.00–0.19). There was strong positive correlation between CHS in AQUAREL and BP in SF-36 and between DYS in AQUAREL and SF in SF-36. CHS and DYS showed statistically significant weak to strong positive correlation to all domains in SF-36. Meanwhile, ARR in AQUAREL showed statistically non-significant very weak positive correlation to GH, RP, VT in SF-36. Furthermore, total AQUAREL and total SF-36 questionnaire showed a statistically significant, moderate positive correlation (r = 0.543; p = 0.000) (Table 2).

**Table 2:** Kendall’s Tau correlation between Indonesian version of AQUAREL and SF-36

	GH	PF	RP	RE	SF	BP	VT	MH	6MWT	NT proBNP
CHS	0.280*	0.376**	0.285*	0.439**	0.516**	0.612**	0.234*	0.373**	0.090	− 0.231*
DYS	0.370**	0.547**	0.370**	0.327*	0.616**	0.486**	0.239*	0.451**	0.228*	− 0.268*
ARR	0.030	0.294*	0.187	0.343*	0.456**	0.489**	0.061	0.329**	− 0.025	− 0.079

* Significant at p < 0.05

** Significant at p < 0.01

#### Correlation between AQUAREL and 6MWT and NT-proBNP

Correlation between AQUAREL and 6MWT and NT-proBNP was assessed by Kendall’s Tau non-parametric test (Additional file 1: Appendix S10). Domain DYS showed weak positive correlation with 6MWT (r = 0.228; p = 0.048), while two others domains did not show any correlation with 6MWT. There was weak negative correlation between CHS and DYS with NT-proBNP. However, total AQUAREL was not statistically correlated with 6MWT (r = 0.122) and NT-proBNP (r = − 0.220) (Table 2).

#### Internal consistency of AQUAREL

Internal consistency was tested by Cronbach-α coefficient and correlation coefficient in which Cronbach-α > 0.70 is considered acceptable. CHS, DYS, ARR, and total AQUAREL have Cronbach-α of 0.70, 0.481, 0.486, and 0.728 respectively.

Inter-item correlations were calculated in each domain. Within CHS (number 1–6, 11, and 12), item number 1–5 showed statistically significant moderate to strong correlation to each other (Additional file 1: Appendix S2). However, item number 11 was not statistically correlated to other items in CHS. Within DYS (number 7–10, 18–20), there were strong positive correlations between item 8 and 9 (r = 0.679), item 7 and 8 (r = 0.622), and item 9 and 10 (r = 0.635) (Additional file 1: Appendix S3). In ARR, (number 13–17), a significant weak positive correlation only found between item number 13 and 14 (Additional file 1: Appendix S4). There were no significant correlations between other items in ARR domain.

Inter-domain correlation was tested by using Kendall Tau nonparametric test (Additional file 1: Appendix S6). Total AQUAREL showed very strong and strong positive correlation with CHS (r = 0.863) and DYS (r = 0.684), respectively. CHS also showed strong positive correlation with DYS (r = 0.684). Meanwhile, correlation between ARR and DYS only showed weak positive correlation (r = 0.331).

#### Repeatability of AQUAREL

Repeatability test was conducted by asking the patients to fill the questionnaire twice. Patients were asked to visit the hospital 7 days after the first meeting.

Item 13 showed a very strong positive correlation between day 1 and 8 (Additional file 1: Appendix S11). On the contrary, items 1, 2, 4, 7, 8, 9, 11, 14, and 19 did not show any significant correlation between day 1 and 8, and item 17 could not have correlation because the data result was constant for all patients in both day 1 and 8. However, when combined as per domain, the ARR, CHS, and DYS showed strong (r = 0.632), moderate (r = 0.493), and weak (r = 0.393) positive correlation for day 1 and 8, respectively (Additional file 1: Appendix S12). For total questionnaire, the correlation between day 1 and 8 showed moderate positive correlation (r = 0.581).

There was no significant difference of Wilcoxon test for repeatability test in 20 patients between day 1 and 8 for CHS, DYS, and ARR (*p* = 0.826; 0.682; 0.717 respectively) (Additional file 1: Appendixes S13, S14). The t-test analysis of total AQUAREL also showed no difference (*p* = 0.834) (Additional file 1: Appendix S15). The results of ICC per domain of CHS, DYS, ARR, and total AQUAREL were 0.698, 0.776, 0.859, and 0.779 respectively (Additional file 1: Appendix S17). The summary of validity-reliability test of Indonesia version of AQUAREL can be seen in Table 3.

**Table 3:** Summary of validity–reliability test of Indonesian version of AQUAREL

	Parameters	Result
Validity	Correlation with SF-36	Strong correlation between CHS–BP and between DYS–SF Moderate correlation between total AQUAREL and total SF-36
	Correlation with 6MWT	Weak correlation between DYS–6MWT
	Correlation with NT-proBNP	Weak correlation between CHS–DYS with NT pro BNP
Reliability	Inter-item correlation	Item 1–5 showed moderate to strong correlation in CHS Item 7–8, 8–9, 9–10 showed strong correlation in DYS
	Inter-domain correlation	Very strong correlation between CHS and total AQUAREL Strong correlation between DYS and total AQUAREL Strong correlation between CHS and DYS
	Day-1 to day-8 comparison	No significant difference mean value Good correlation (Kendall Tau) of total AQUAREL Good correlation (ICC) total AQUAREL

### Discussion

Indonesian-AQUAREL consists of 20 item questions, which were derived from 3 domains: Chest Discomfort, Dyspnea and Exertion, and Arrhythmia. The structure of Indonesian version followed the English version of AQUAREL. Each question was followed by 5 multiple choices. Up to our knowledge, this is the second formal publication of AQUAREL questionnaire adaptation after Portuguese [15].

Domains in Indonesian-AQUAREL have significant weak to strong correlation with domains in SF-36, except ARR domain, which showed no significant correlation with GH, RP, and VT domains in SF-36. However, another study in Portugal showed all domains in AQUAREL and SF-36 showed significant weak to strong positive correlation. This might happen because of patient’s different understanding of the questionnaire, they might not consider their complaints as a significant part of their quality of life. Overall, Indonesian-AQUAREL and SF-36 showed significant moderate positive correlation. Six minutes walking test, the objective parameter to assess functional capacity [16], only showed weak positive correlation with DYS. Weak negative correlation was found between NT-proBNP as the quantitative markers of heart failure [17], with DYS and CHS. This might happen as dyspnea and chest discomfort are neither sensitive nor specific for cardiac diseases [18, 19]. Indonesian-AQUAREL did not show any correlation with 6MWT and NT-proBNP. Oliveira et al. also did not found any significant correlation between AQUAREL and 6MWT in patients with permanent pacemaker [15].

The whole Indonesian-AQUAREL has good internal consistency (Cronbach-α > 0.7). However, Cronbach-α coefficient for both DYS and ARR were < 0.7. This finding was in accordance with study in Portugal which also showed similar results [15].

In CHS domain, inter-item correlation varied from moderate to strong, except item number 6, 11, and 12 which showed no correlation with most of other items in same domain. We assumed that many patients have difficulty to differentiate questions in item number 6, 11, and 12. In DYS domain, most items have weak to strong correlation, except item number 18 and 20 which show no correlation with other items. In ARR domain, only item number 13 and 14 showed correlation. We assumed patients did not considered arrhythmia as a problem that interferes their quality of life.

Inter-domain correlation showed weak to strong correlation. There were no other studies which assessed either inter-items or inter-domains correlation in AQUAREL questionnaire. Most of other studies assessed internal consistency based on Cronbach-α coefficient in each domain.

Even though Indonesian-AQUAREL has good internal consistency, some items were not correlated to each other. This might affect the ability of the questionnaire to discriminate the quality of life in some sub-populations of patients. Combination with Indonesian version of SF-36 is needed if the questionnaire wants to be used in Indonesian culture setting. The difference of correlation coefficient between inter-item, inter-domain, and total questionnaire was assumed because of the statistical method difference used to analyze the correlation (parametric and non-parametric).

Repeatability of Indonesian-AQUAREL was considered good with no significant difference between day 1 and 8. This finding was in accordance with other study which showed moderate to strong repeatability coefficient, assessed by t-test. Oliveira et al. also did not get any significant difference result between domains, which was repeated in 1–2 weeks’ period [15].

The ICC of total AQUAREL both day 1 and 8 showed that CHS has moderate reliability and DYS, ARR, and total AQUAREL has good reliability [20].

### Conclusion

In conclusion, the Indonesian version of AQUAREL has good convergent and discriminant validity, as well as good reliability. It’s easily applicable in clinical setting due to its simple nature and comprehensible test. Thus, it could be used as a specific questionnaire to assess quality of life in permanent pacemaker patients.

## Limitation

This study has few limitations. First, we did not evaluate the correlation between the receptiveness of the patients with their education level and ethnical background. Our samples were also very homogenous and from one center only, prompting the need to validate our questionnaire in other patient’s groups.

## Additional file

**Additional file 1: Appendix S1.** Normality test for each item in AQUAREL Questionnaire. SPSS result of the normality test for each item in AQUAREL. Questionnaire using Kolmagorov Smirnov and Saphiro-Wilk test. **Appendix S2.** Kendall’s Tau correlation inter item in *Chest discomfort* domain. SPSS result of item to item item correlation in chest discomfort domain using Kendall’s Tau correlation test. **Appendix S3.** Kendall’s Tau correlation inter item in *Dyspnea* domain. SPSS result of item to item correlation in dyspnea domain using Kendall’s Tau correlation test. **Appendix S4.** Kendall’s Tau correlation inter item in *Arrhythmia* domain. SPSS result of item to item correlation in arrhythmia domain using Kendall’s Tau correlation test. **Appendix S5.** Inter domain normality test in AQUAREL questionnaire. SPSS result of the normality test for inter domain (Chest discomfort, dyspnea, arrhythmia) in AQUAREL Questionnaire using Kolmagorov Smirnov and Saphiro-Wilk test. **Appendix S6.** Inter domain correlation test in AQUAREL questionnaire. SPSS result of inter domain correlation of AQUAREL questionnaire using Kendall’s tau correlation test. **Appendix S7.** Kendall’s Tau correlation between 3 domain AQUAREL and 8 domain SF-36. SPSS result of correlation between 3 domain AQUAREL and 8 domain SF-36 using Kendall’s Tau correlation test. **Appendix S8.** Kendall Tau’s correlation between total SF-36 and total AQUAREL. SPSS result of correlation between total SF-36 and total AQUAREL using Kendall’s Tau correlation test. **Appendix S9.** Kendall’s Tau correlation between Domain SF-36 and 6MWT and NT pro-BNP. SPSS result of correlation between Domain SF-36 and 6MWT dan NT pro-BNP using Kendall’s tau correlation test. **Appendix S10.** Kendall’s Tau correlation between AQUAREL and 6MWT and NT pro-BNP. SPSS result of correlation between AQUAREL and 6MWT and NT pro-BNP using Kendall’s tau correlation test. **Appendix S11.** Kendall’s Tau Correlation Inter Item AQUAREL Day-1 and Day-8. SPSS result of inter item correlation of AQUAREL Day-1 and Day-8 (repeat) using Kendall’s Tau Correlation test. **Appendix S12.** Kendall’s Tau Correlation Inter domain AQUAREL Day-1 and Day-8. SPSS result of inter domain correlation of AQUAREL Day-1 and Day-8 (repeat) using Kendall’s Tau Correlation test. **Appendix S13.** Wilcoxon's Signed Rank Test Inter Item AQUAREL Day 1 and 8. The comparison of Inter Item Wilcoxon’s signed rank in AQUAREL between day 1 and 8. **Appendix S14.** Wilcoxon’s Signed Rank Inter Domain AQUAREL Day 1 and 8. The Wilcoxon’s Signed Rank of Inter domain of AQUAREL between day 1 and 8.** Appendix S15.** T-Test Analysis of Total AQUAREL Day 1 and 8. Total AQUAREL of day 1 and 8 with t-test analysis. **Appendix S16.** Kendall’s Tau Correlation Inter Item AQUAREL Questionnaire. SPSS result of item to item (20 items) correlation of AQUAREL Questionnaire. **Appendix S17.** ICC between Day 1 and Day 8 by Domain and Total AQUAREL. SPSS result of intraclass correlation coefficient (ICC) analysis of AQUAREL three domains and comparison of total AQUAREL in day 1 with day 8. **Appendix S18.** AQUAREL Questionnaire. The English version of AQUAREL Questionnaire that is translated and validated into the Indonesian version of AQUAREL Questionnaire. **Appendix S19.** Initial English-Indonesia AQUAREL Translation 1. AQUAREL questionnaire translated to Bahasa Indonesia by the translator with medical background. **Appendix S20.** Initial English-Indonesia AQUAREL Translation 2. AQUAREL questionnaire translated to Bahasa Indonesia by the translator with non-medical background. **Appendix S21.** Translation Synthesis AQUAREL Questionnaire. The final Indonesian version of AQUAREL questionnaire. **Appendix S22.** Back Translation AQUAREL Questioannaire 1. The Indonesian version of AQUAREL is translated back to English language by a native English speaker 1. **Appendix S23.** Back Translation AQUAREL Questioannaire 2. The Indonesian version of AQUAREL is translated back to English language by a different native English speaker.

## Abbreviations

AQUAREL: Assessment of Quality of Life and Related Events
6MWT: 6-min walking test
NYHA: New York Heart Association
ARR: arrhythmia
DYS: dyspnea on effort
CHS: chest discomfort
GH: general health
PF: physical functioning
RP: role-physical
BP: bodily pain
VT: vitality
SF: social functioning
RE: role-emotional
MH: mental health
