# Supplementary material for: Cross-cultural adaptation and validation of the Indonesian version of AQUAREL on patients with permanent pacemaker: a cross-sectional study
Source: BMC Res Notes. 2019 Mar 28;12:178. doi: 10.1186/s13104-019-4208-1 (PMC6437905; doi:10.1186/s13104-019-4208-1)
Supplement: Supplementary file 1 — Additional file 1: Appendix S1. Normality test for each item in AQUAREL Questionnaire. SPSS result of the normality test for each item in AQUAREL. Questionnaire using Kolmagorov Smirnov and Saphiro-Wilk test. Appendix S2. Kendall’s Tau correlation inter item in Chest discomfort domain. SPSS result of item to item item correlation in chest discomfort domain using Kendall’s Tau correlation test. Appendix S3. Kendall’s Tau correlation inter item in Dyspnea domain. SPSS result of item to item correlation in dyspnea domain using Kendall’s Tau correlation test. Appendix S4. Kendall’s Tau correlation inter item in Arrhythmia domain. SPSS result of item to item correlation in arrhythmia domain using Kendall’s Tau correlation test. Appendix S5. Inter domain normality test in AQUAREL questionnaire. SPSS result of the normality test for inter domain (Chest discomfort, dyspnea, arrhythmia) in AQUAREL Questionnaire using Kolmagorov Smirnov and Saphiro-Wilk test. Appendix S6. Inter domain correlation test in AQUAREL questionnaire. SPSS result of inter domain correlation of AQUAREL questionnaire using Kendall’s tau correlation test. Appendix S7. Kendall’s Tau correlation between 3 domain AQUAREL and 8 domain SF-36. SPSS result of correlation between 3 domain AQUAREL and 8 domain SF-36 using Kendall’s Tau correlation test. Appendix S8. Kendall Tau’s correlation between total SF-36 and total AQUAREL. SPSS result of correlation between total SF-36 and total AQUAREL using Kendall’s Tau correlation test. Appendix S9. Kendall’s Tau correlation between Domain SF-36 and 6MWT and NT pro-BNP. SPSS result of correlation between Domain SF-36 and 6MWT dan NT pro-BNP using Kendall’s tau correlation test. Appendix S10. Kendall’s Tau correlation between AQUAREL and 6MWT and NT pro-BNP. SPSS result of correlation between AQUAREL and 6MWT and NT pro-BNP using Kendall’s tau correlation test. Appendix S11. Kendall’s Tau Correlation Inter Item AQUAREL Day-1 and Day-8. SPSS result of inter item correla [file 13104_2019_4208_MOESM1_ESM.docx]

**APPENDIX S1**

**Normality test for each item in AQUAREL Questionnaire**

|  | Kolmogorov-Smirnov^a^ | | | Shapiro-Wilk | | |
| --- | --- | --- | --- | --- | --- | --- |
|  | Statistic | df | Sig. | Statistic | df | Sig. |
| item1 | ,285 | 40 | ,000 | ,796 | 40 | ,000 |
| item2 | ,266 | 40 | ,000 | ,796 | 40 | ,000 |
| item3 | ,307 | 40 | ,000 | ,768 | 40 | ,000 |
| item4 | ,326 | 40 | ,000 | ,711 | 40 | ,000 |
| item5 | ,306 | 40 | ,000 | ,751 | 40 | ,000 |
| item6 | ,398 | 40 | ,000 | ,667 | 40 | ,000 |
| item7 | ,232 | 40 | ,000 | ,836 | 40 | ,000 |
| item8 | ,328 | 40 | ,000 | ,711 | 40 | ,000 |
| item9 | ,395 | 40 | ,000 | ,632 | 40 | ,000 |
| item10 | ,369 | 40 | ,000 | ,704 | 40 | ,000 |
| item11 | ,511 | 40 | ,000 | ,428 | 40 | ,000 |
| item12 | ,479 | 40 | ,000 | ,519 | 40 | ,000 |
| item13 | ,512 | 40 | ,000 | ,395 | 40 | ,000 |
| item14 | ,286 | 40 | ,000 | ,767 | 40 | ,000 |
| item15 | ,465 | 40 | ,000 | ,541 | 40 | ,000 |
| item16 | ,389 | 40 | ,000 | ,662 | 40 | ,000 |
| item17 | ,540 | 40 | ,000 | ,229 | 40 | ,000 |
| item18 | ,531 | 40 | ,000 | ,291 | 40 | ,000 |
| item19 | ,326 | 40 | ,000 | ,697 | 40 | ,000 |
| item20 | ,212 | 40 | ,000 | ,891 | 40 | ,001 |
| a. Lilliefors Significance Correction | | | | | | |

**APPENDIX S2**

**Kendall’s Tau correlation inter item in *Chest discomfort* domain**

|  | | | item1 | item2 | item3 | item4 | item5 | item6 | item11 | item12 | |
| --- | --- | --- | --- | --- | --- | --- | --- | --- | --- | --- | --- |
| Kendall's tau_b | item1 | Correlation Coefficient | 1,000 | ,509^**^ | ,549^**^ | ,544^**^ | ,465^**^ | ,444^**^ | ,080 | ,247 |  |
|  |  | Sig. (2-tailed) |  | ,000 | ,000 | ,000 | ,001 | ,002 | ,596 | ,092 |  |
|  |  | N | 40 | 40 | 40 | 40 | 40 | 40 | 40 | 40 |  |
|  | item2 | Correlation Coefficient | ,509^**^ | 1,000 | ,717^**^ | ,619^**^ | ,490^**^ | ,258 | -,012 | ,129 |  |
|  |  | Sig. (2-tailed) | ,000 |  | ,000 | ,000 | ,000 | ,073 | ,936 | ,372 |  |
|  |  | N | 40 | 40 | 40 | 40 | 40 | 40 | 40 | 40 |  |
|  | item3 | Correlation Coefficient | ,549^**^ | ,717^**^ | 1,000 | ,756^**^ | ,620^**^ | ,269 | -,006 | ,353^*^ |  |
|  |  | Sig. (2-tailed) | ,000 | ,000 |  | ,000 | ,000 | ,062 | ,967 | ,014 |  |
|  |  | N | 40 | 40 | 40 | 40 | 40 | 40 | 40 | 40 |  |
|  | item4 | Correlation Coefficient | ,544^**^ | ,619^**^ | ,756^**^ | 1,000 | ,705^**^ | ,276 | ,083 | ,374^*^ |  |
|  |  | Sig. (2-tailed) | ,000 | ,000 | ,000 |  | ,000 | ,059 | ,581 | ,011 |  |
|  |  | N | 40 | 40 | 40 | 40 | 40 | 40 | 40 | 40 |  |
|  | item5 | Correlation Coefficient | ,465^**^ | ,490^**^ | ,620^**^ | ,705^**^ | 1,000 | ,388^**^ | ,003 | ,454^**^ |  |
|  |  | Sig. (2-tailed) | ,001 | ,000 | ,000 | ,000 |  | ,008 | ,983 | ,002 |  |
|  |  | N | 40 | 40 | 40 | 40 | 40 | 40 | 40 | 40 |  |
|  | item6 | Correlation Coefficient | ,444^**^ | ,258 | ,269 | ,276 | ,388^**^ | 1,000 | ,225 | ,264 |  |
|  |  | Sig. (2-tailed) | ,002 | ,073 | ,062 | ,059 | ,008 |  | ,149 | ,082 |  |
|  |  | N | 40 | 40 | 40 | 40 | 40 | 40 | 40 | 40 |  |
|  | item11 | Correlation Coefficient | ,080 | -,012 | -,006 | ,083 | ,003 | ,225 | 1,000 | ,288 |  |
|  |  | Sig. (2-tailed) | ,596 | ,936 | ,967 | ,581 | ,983 | ,149 |  | ,065 |  |
|  |  | N | 40 | 40 | 40 | 40 | 40 | 40 | 40 | 40 |  |
|  | item12 | Correlation Coefficient | ,247 | ,129 | ,353^*^ | ,374^*^ | ,454^**^ | ,264 | ,288 | 1,000 |  |
|  |  | Sig. (2-tailed) | ,092 | ,372 | ,014 | ,011 | ,002 | ,082 | ,065 |  |  |
|  |  | N | 40 | 40 | 40 | 40 | 40 | 40 | 40 | 40 |  |
| **. Correlation is significant at the 0.01 level (2-tailed). | | | | | | | | | | |  |
| *. Correlation is significant at the 0.05 level (2-tailed). | | | | | | | | | | |  |

**APPENDIX S3**

**Kendall’s Tau correlation inter item in *Dyspnea* domain**

|  | | | item7 | item8 | item9 | item10 | item18 | item19 | item20 |
| --- | --- | --- | --- | --- | --- | --- | --- | --- | --- |
| Kendall's tau_b | item7 | Correlation Coefficient | 1,000 | ,622^**^ | ,469^**^ | ,355^*^ | ,012 | ,102 | ,182 |
|  |  | Sig. (2-tailed) |  | ,000 | ,001 | ,013 | ,935 | ,468 | ,177 |
|  |  | N | 40 | 40 | 40 | 40 | 40 | 40 | 40 |
|  | item8 | Correlation Coefficient | ,622^**^ | 1,000 | ,679^**^ | ,408^**^ | -,222 | ,054 | ,034 |
|  |  | Sig. (2-tailed) | ,000 |  | ,000 | ,006 | ,141 | ,709 | ,805 |
|  |  | N | 40 | 40 | 40 | 40 | 40 | 40 | 40 |
|  | item9 | Correlation Coefficient | ,469^**^ | ,679^**^ | 1,000 | ,635^**^ | -,186 | ,206 | ,056 |
|  |  | Sig. (2-tailed) | ,001 | ,000 |  | ,000 | ,225 | ,160 | ,690 |
|  |  | N | 40 | 40 | 40 | 40 | 40 | 40 | 40 |
|  | item10 | Correlation Coefficient | ,355^*^ | ,408^**^ | ,635^**^ | 1,000 | ,104 | ,305^*^ | ,028 |
|  |  | Sig. (2-tailed) | ,013 | ,006 | ,000 |  | ,497 | ,037 | ,845 |
|  |  | N | 40 | 40 | 40 | 40 | 40 | 40 | 40 |
|  | item18 | Correlation Coefficient | ,012 | -,222 | -,186 | ,104 | 1,000 | ,004 | ,227 |
|  |  | Sig. (2-tailed) | ,935 | ,141 | ,225 | ,497 |  | ,978 | ,116 |
|  |  | N | 40 | 40 | 40 | 40 | 40 | 40 | 40 |
|  | item19 | Correlation Coefficient | ,102 | ,054 | ,206 | ,305^*^ | ,004 | 1,000 | ,077 |
|  |  | Sig. (2-tailed) | ,468 | ,709 | ,160 | ,037 | ,978 |  | ,578 |
|  |  | N | 40 | 40 | 40 | 40 | 40 | 40 | 40 |
|  | item20 | Correlation Coefficient | ,182 | ,034 | ,056 | ,028 | ,227 | ,077 | 1,000 |
|  |  | Sig. (2-tailed) | ,177 | ,805 | ,690 | ,845 | ,116 | ,578 |  |
|  |  | N | 40 | 40 | 40 | 40 | 40 | 40 | 40 |
| **. Correlation is significant at the 0.01 level (2-tailed). | | | | | | | | | |
| *. Correlation is significant at the 0.05 level (2-tailed). | | | | | | | | | |

**APPENDIX S4**

**Kendall’s Tau correlation inter item in *Arrhythmia* domain**

|  | | | item13 | item14 | item15 | item16 | item17 |
| --- | --- | --- | --- | --- | --- | --- | --- |
| Kendall's tau_b | item13 | Correlation Coefficient | 1,000 | ,314^*^ | -,047 | ,076 | -,085 |
|  |  | Sig. (2-tailed) |  | ,036 | ,758 | ,618 | ,589 |
|  |  | N | 40 | 40 | 40 | 40 | 40 |
|  | item14 | Correlation Coefficient | ,314^*^ | 1,000 | ,275 | ,222 | -,076 |
|  |  | Sig. (2-tailed) | ,036 |  | ,063 | ,128 | ,617 |
|  |  | N | 40 | 40 | 40 | 40 | 40 |
|  | item15 | Correlation Coefficient | -,047 | ,275 | 1,000 | ,238 | -,119 |
|  |  | Sig. (2-tailed) | ,758 | ,063 |  | ,112 | ,444 |
|  |  | N | 40 | 40 | 40 | 40 | 40 |
|  | item16 | Correlation Coefficient | ,076 | ,222 | ,238 | 1,000 | 0,000 |
|  |  | Sig. (2-tailed) | ,618 | ,128 | ,112 |  | 1,000 |
|  |  | N | 40 | 40 | 40 | 40 | 40 |
|  | item17 | Correlation Coefficient | -,085 | -,076 | -,119 | 0,000 | 1,000 |
|  |  | Sig. (2-tailed) | ,589 | ,617 | ,444 | 1,000 |  |
|  |  | N | 40 | 40 | 40 | 40 | 40 |

**APPENDIX S5**

**Inter domain normality test in AQUAREL questionnaire**

|  | Kolmogorov-Smirnov^a^ | | | Shapiro-Wilk | | |
| --- | --- | --- | --- | --- | --- | --- |
|  | Statistic | df | Sig. | Statistic | df | Sig. |
| chs | ,159 | 40 | ,013 | ,871 | 40 | ,000 |
| dys | ,106 | 40 | ,200^*^ | ,958 | 40 | ,141 |
| arr | ,175 | 40 | ,003 | ,878 | 40 | ,000 |
| totalAQUAREL | ,094 | 40 | ,200^*^ | ,953 | 40 | ,097 |
| *. This is a lower bound of the true significance. | | | | | | |
| a. Lilliefors Significance Correction | | | | | | |

**APPENDIX S6**

**Inter domain correlation test in AQUAREL questionnaire**

|  | | | chs | dys | Arr | totalA |
| --- | --- | --- | --- | --- | --- | --- |
| Kendall's tau_b | chs | Correlation Coefficient | 1,000 | ,684^**^ | ,402^**^ | ,863^**^ |
|  |  | Sig. (2-tailed) |  | ,000 | ,001 | ,000 |
|  |  | N | 40 | 40 | 40 | 40 |
|  | dys | Correlation Coefficient | ,684^**^ | 1,000 | ,331^**^ | ,789^**^ |
|  |  | Sig. (2-tailed) | ,000 |  | ,007 | ,000 |
|  |  | N | 40 | 40 | 40 | 40 |
|  | arr | Correlation Coefficient | ,402^**^ | ,331^**^ | 1,000 | ,481^**^ |
|  |  | Sig. (2-tailed) | ,001 | ,007 |  | ,000 |
|  |  | N | 40 | 40 | 40 | 40 |
|  | totalA | Correlation Coefficient | ,863^**^ | ,789^**^ | ,481^**^ | 1,000 |
|  |  | Sig. (2-tailed) | ,000 | ,000 | ,000 |  |
|  |  | N | 40 | 40 | 40 | 40 |
| **. Correlation is significant at the 0.01 level (2-tailed). | | | | | | |

**APPENDIX S7**

**Kendall’s Tau correlation between 3 domain AQUAREL and 8 domain SF-36**

|  |  |  | gh | pf | | Rp | re | | sf | bp | vt | | mh |
| --- | --- | --- | --- | --- | --- | --- | --- | --- | --- | --- | --- | --- | --- |
| Kendall's tau_b | chs | Correlation Coefficient | ,280^*^ | ,376^**^ | ,285^*^ | | | ,439^**^ | ,516^**^ | ,612^**^ | ,234^*^ | ,373^**^ | |
|  |  | Sig. (2-tailed) | ,019 | ,001 | ,025 | | | ,001 | ,000 | ,000 | ,047 | ,002 | |
|  |  | N | 40 | 40 | 40 | | | 40 | 40 | 40 | 40 | 40 | |
|  | dys | Correlation Coefficient | ,370^**^ | ,547^**^ | ,370^**^ | | | ,327^*^ | ,616^**^ | ,486^**^ | ,239^*^ | ,451^**^ | |
|  |  | Sig. (2-tailed) | ,002 | ,000 | ,004 | | | ,013 | ,000 | ,000 | ,044 | ,000 | |
|  |  | N | 40 | 40 | 40 | | | 40 | 40 | 40 | 40 | 40 | |
|  | Arr | Correlation Coefficient | ,030 | ,294^*^ | ,187 | | | ,343^*^ | ,456^**^ | ,489^**^ | ,061 | ,329^**^ | |
|  |  | Sig. (2-tailed) | ,808 | ,015 | ,156 | | | ,011 | ,000 | ,000 | ,614 | ,008 | |
|  |  | N | 40 | 40 | 40 | | | 40 | 40 | 40 | 40 | 40 | |
| *. Correlation is significant at the 0.05 level (2-tailed). | | | | | | | | | | |  | |  |
| **. Correlation is significant at the 0.01 level (2-tailed). | | | | | | | | | | |  | |  |

**APPENDIX S8**

**Kendall Tau’s correlation between total SF-36 and total AQUAREL**

|  | | | ttlSF | ttlA |
| --- | --- | --- | --- | --- |
| Kendall's tau_b | ttlSF | Correlation Coefficient | 1,000 | ,543^**^ |
|  |  | Sig. (2-tailed) | . | ,000 |
|  |  | N | 40 | 40 |
|  | ttlA | Correlation Coefficient | ,543^**^ | 1,000 |
|  |  | Sig. (2-tailed) | ,000 | . |
|  |  | N | 40 | 40 |

**APPENDIX S9**

**Kendall’s Tau correlation between Domain SF-36 and 6MWT dan NT pro-BNP**

|  | | | gh | pf | rp | re | sf | bp | vt | mh | mwt | nt |
| --- | --- | --- | --- | --- | --- | --- | --- | --- | --- | --- | --- | --- |
| Kendall's tau_b | Gh | r | 1,000 | ,282^*^ | ,240 | ,082 | ,422^**^ | ,031 | ,311^**^ | ,308^*^ | ,173 | -,269^*^ |
|  |  | ρ |  | ,018 | ,062 | ,534 | ,001 | ,809 | ,009 | ,011 | ,135 | ,020 |
|  |  | N | 40 | 40 | 40 | 40 | 40 | 40 | 40 | 40 | 40 | 40 |
|  | Pf | r | ,282^*^ | 1,000 | ,489^**^ | ,376^**^ | ,463^**^ | ,394^**^ | ,354^**^ | ,231 | ,363^**^ | -,179 |
|  |  | ρ | ,018 |  | ,000 | ,003 | ,000 | ,002 | ,002 | ,053 | ,001 | ,114 |
|  |  | N | 40 | 40 | 40 | 40 | 40 | 40 | 40 | 40 | 40 | 40 |
|  | Rp | r | ,240 | ,489^**^ | 1,000 | ,574^**^ | ,414^**^ | ,335^*^ | ,146 | ,185 | ,028 | -,131 |
|  |  | ρ | ,062 | ,000 |  | ,000 | ,002 | ,013 | ,248 | ,152 | ,822 | ,288 |
|  |  | N | 40 | 40 | 40 | 40 | 40 | 40 | 40 | 40 | 40 | 40 |
|  | Re | r | ,082 | ,376^**^ | ,574^**^ | 1,000 | ,290^*^ | ,614^**^ | ,164 | ,244 | -,002 | -,231 |
|  |  | ρ | ,534 | ,003 | ,000 |  | ,035 | ,000 | ,205 | ,063 | ,989 | ,065 |
|  |  | N | 40 | 40 | 40 | 40 | 40 | 40 | 40 | 40 | 40 | 40 |
|  | Sf | r | ,422^**^ | ,463^**^ | ,414^**^ | ,290^*^ | 1,000 | ,416^**^ | ,269^*^ | ,445^**^ | ,039 | -,160 |
|  |  | ρ | ,001 | ,000 | ,002 | ,035 |  | ,002 | ,032 | ,000 | ,747 | ,188 |
|  |  | N | 40 | 40 | 40 | 40 | 40 | 40 | 40 | 40 | 40 | 40 |
|  | Bp | r | ,031 | ,394^**^ | ,335^*^ | ,614^**^ | ,416^**^ | 1,000 | ,101 | ,302^*^ | ,123 | -,199 |
|  |  | ρ | ,809 | ,002 | ,013 | ,000 | ,002 |  | ,420 | ,017 | ,310 | ,100 |
|  |  | N | 40 | 40 | 40 | 40 | 40 | 40 | 40 | 40 | 40 | 40 |
|  | Vt | r | ,311^**^ | ,354^**^ | ,146 | ,164 | ,269^*^ | ,101 | 1,000 | ,360^**^ | ,092 | -,140 |
|  |  | ρ | ,009 | ,002 | ,248 | ,205 | ,032 | ,420 |  | ,003 | ,419 | ,218 |
|  |  | N | 40 | 40 | 40 | 40 | 40 | 40 | 40 | 40 | 40 | 40 |
|  | Mh | r | ,308^*^ | ,231 | ,185 | ,244 | ,445^**^ | ,302^*^ | ,360^**^ | 1,000 | ,077 | -,271^*^ |
|  |  | ρ | ,011 | ,053 | ,152 | ,063 | ,000 | ,017 | ,003 |  | ,508 | ,019 |
|  |  | N | 40 | 40 | 40 | 40 | 40 | 40 | 40 | 40 | 40 | 40 |
|  | mwt | r | ,173 | ,363^**^ | ,028 | -,002 | ,039 | ,123 | ,092 | ,077 | 1,000 | -,214 |
|  |  | ρ | ,135 | ,001 | ,822 | ,989 | ,747 | ,310 | ,419 | ,508 |  | ,053 |
|  |  | N | 40 | 40 | 40 | 40 | 40 | 40 | 40 | 40 | 40 | 40 |
|  | Nt | r | -,269^*^ | -,179 | -,131 | -,231 | -,160 | -,199 | -,140 | -,271^*^ | -,214 | 1,000 |
|  |  | ρ | ,020 | ,114 | ,288 | ,065 | ,188 | ,100 | ,218 | ,019 | ,053 |  |
|  |  | N | 40 | 40 | 40 | 40 | 40 | 40 | 40 | 40 | 40 | 40 |

**p<0,01

* p<0,05

**APPENDIX S10**

**Kendall’s Tau correlation between AQUAREL and 6MWT and NT pro-BNP**

|  |  |  | chs | dys | arr | totalA |
| --- | --- | --- | --- | --- | --- | --- |
| Kendall's Tau_b | mwt | Correlation Coefficient | ,090 | ,228^*^ | -,025 | ,122 |
|  |  | Sig. (2-tailed) | ,431 | ,048 | ,830 | ,273 |
|  |  | N | 40 | 40 | 40 | 40 |
|  | nt | Correlation Coefficient | -,231^*^ | -,268^*^ | -,079 | -,224^*^ |
|  |  | Sig. (2-tailed) | ,043 | ,020 | ,504 | ,045 |
|  |  | N | 40 | 40 | 40 | 40 |
| **. Correlation is significant at the 0.01 level (2-tailed). | | | | |  |  |
| *. Correlation is significant at the 0.05 level (2-tailed). | | | | |  |  |

**APPENDIX S11**

**Kendall’s Tau Correlation Inter Item AQUAREL Day-1 and Day-8**

|  | Koefisien korelasi | P |
| --- | --- | --- |
| item1a - item1b | 0,127 | 0,537 |
| item2a - item2b | 0,331 | 0,099 |
| item3a - item3b | 0,579^**^ | 0,004 |
| item4a - item4b | 0,258 | 0,210 |
| item5a - item5b | 0,531^*^ | 0,011 |
| item6a - item6b | 0,505^*^ | 0,020 |
| item7a - item7b | 0,298 | 0,133 |
| item8a - item8b | 0,325 | 0,121 |
| item9a - item9b | 0,422 | 0,051 |
| item10a - item10b | 0,515^*^ | 0,016 |
| item11a - item11b | 0,216 | 0,347 |
| item12a - item12b | 0,458^*^ | 0,037 |
| item13a - item13b | 0,813^**^ | 0,000 |
| item14a - item14b | 0,306 | 0,138 |
| item15a - item15b | 0,668^**^ | 0,003 |
| item16a - item16b | 0,644^**^ | 0,003 |
| item17a - item17b | . | . |
| item18a - item18b | 0,641^**^ | 0,005 |
| item19a - item19b | 0,258 | 0,213 |
| item20a - item20b | 0,570^**^ | 0,003 |

(a : hari-1; b : hari-8)

** p<0,01

*p<0,05

**APPENDIX S12**

**Kendall’s Tau Correlation Inter Domain AQUAREL Day-1 and Day-8**

|  | Koefisien korelasi | P |
| --- | --- | --- |
| chs1 - chs2 | 0,493^**^ | 0,004 |
| dys1 - dys2 | 0,393^*^ | 0,025 |
| arr1 - arr2 | 0,632^**^ | 0,001 |

chs1 : domain *chest discomfort* pada hari-1; chs2 : domain *chest discomfort* pada hari-8;

dys1 : domain *dyspneu* pada hari-1; dys2 : domain *dyspneu* pada hari-8;

arr1 : domain *arrythmia* pada hari-1; arr2 : domain *arrythmia* pada hari-8;

** p<0,01

*p<0,05

**APPENDIX S13**

**Wilcoxon’s Signed Rank Test Inter Item AQUAREL Day 1 and 8**

|  | **Negative Ranks** | **Positive Ranks** | **Ties** | **P** |  | **Negative Ranks** | **Positive Ranks** | **Ties** | **P** |
| --- | --- | --- | --- | --- | --- | --- | --- | --- | --- |
| item 1 | 3 | 7 | 10 | 0.174 | item 11 | 2 | 2 | 16 | 1.000 |
| item 2 | 5 | 5 | 10 | 0.716 | item 12 | 2 | 3 | 15 | 0.783 |
| item 3 | 3 | 5 | 12 | 0.351 | item 13 | 0 | 1 | 19 | 0.317 |
| item 4 | 6 | 5 | 9 | 0.417 | item 14 | 7 | 3 | 10 | 0.265 |
| item 5 | 6 | 2 | 12 | 0.107 | item 15 | 3 | 2 | 15 | 0.655 |
| item 6 | 2 | 4 | 14 | 0.739 | item 16 | 1 | 6 | 13 | 0.053 |
| item 7 | 8 | 5 | 7 | 0.637 | item 17 | 2 | 0 | 18 | 0.157 |
| item 8 | 2 | 6 | 12 | 0.176 | item 18 | 1 | 1 | 18 | 0.655 |
| item 9 | 4 | 4 | 12 | 0.773 | item 19 | 2 | 5 | 13 | 0.262 |
| item 10 | 4 | 2 | 14 | 0.414 | item 20 | 4 | 4 | 12 | 0.566 |

**APPENDIX S14**

**Wilcoxon’s Signed Rank Inter Domain AQUAREL Day 1 and 8**

|  | **Negative Ranks** | **Positive Ranks** | **Ties** | **p** |
| --- | --- | --- | --- | --- |
| CHS | 7 | 7 | 6 | 0,826 |
| DYS | 7 | 7 | 6 | 0,682 |
| ARR | 8 | 7 | 5 | 0,717 |

**APPENDIX S15**

**T-Test Analysis of Total AQUAREL Day 1 and 8**

|  | **Day 1** | **Day 8** | **P** |
| --- | --- | --- | --- |
| Total AQUAREL | 87,20 ± 9,192 | 87,60 ± 9,162 | 0,834 |

| **APPENDIX S16**  **Kendall’s Tau Correlation Inter Item AQUAREL Questionnaire** | | | | | | | | | | | | | | | | | | | | | | | |
| --- | --- | --- | --- | --- | --- | --- | --- | --- | --- | --- | --- | --- | --- | --- | --- | --- | --- | --- | --- | --- | --- | --- | --- |
|  | | | item1 | item2 | item3 | item4 | item5 | item6 | item7 | item8 | item9 | item10 | item11 | item12 | item13 | item14 | item15 | item16 | item17 | item18 | item19 | item20 |  |
| Kendall's tau_b | Item 1 | Correlation Coefficient | 1.000 | ,509^**^ | ,549^**^ | ,544^**^ | ,465^**^ | ,444^**^ | ,434^**^ | .276 | ,390^**^ | .259 | .080 | .247 | -.120 | ,320^*^ | .008 | ,338^*^ | .101 | .124 | ,346^*^ | ,282^*^ |  |
|  |  | Sig. (2-tailed) |  | .000 | .000 | .000 | .001 | .002 | .002 | .053 | .007 | .074 | .596 | .092 | .416 | .024 | .959 | .020 | .504 | .407 | .015 | .039 |  |
|  |  | N | 40 | 40 | 40 | 40 | 40 | 40 | 40 | 40 | 40 | 40 | 40 | 40 | 40 | 40 | 40 | 40 | 40 | 40 | 40 | 40 |  |
|  | Item 2 | Correlation Coefficient | ,509^**^ | 1.000 | ,717^**^ | ,619^**^ | ,490^**^ | .258 | ,718^**^ | ,544^**^ | ,443^**^ | ,403^**^ | -.012 | .129 | -.076 | ,333^*^ | -.057 | .246 | .059 | .064 | .245 | .169 |  |
|  |  | Sig. (2-tailed) | .000 |  | .000 | .000 | .000 | .073 | .000 | .000 | .002 | .005 | .936 | .372 | .603 | .018 | .695 | .085 | .693 | .663 | .081 | .210 |  |
|  |  | N | 40 | 40 | 40 | 40 | 40 | 40 | 40 | 40 | 40 | 40 | 40 | 40 | 40 | 40 | 40 | 40 | 40 | 40 | 40 | 40 |  |
|  | Item 3 | Correlation Coefficient | ,549^**^ | ,717^**^ | 1.000 | ,756^**^ | ,620^**^ | .269 | ,505^**^ | ,470^**^ | ,513^**^ | ,406^**^ | -.006 | ,353^*^ | .171 | ,354^*^ | -.227 | ,393^**^ | .150 | .229 | ,278^*^ | ,267^*^ |  |
|  |  | Sig. (2-tailed) | .000 | .000 |  | .000 | .000 | .062 | .000 | .001 | .000 | .005 | .967 | .014 | .240 | .011 | .116 | .006 | .312 | .118 | .048 | .047 |  |
|  |  | N | 40 | 40 | 40 | 40 | 40 | 40 | 40 | 40 | 40 | 40 | 40 | 40 | 40 | 40 | 40 | 40 | 40 | 40 | 40 | 40 |  |
|  | Item 4 | Correlation Coefficient | ,544^**^ | ,619^**^ | ,756^**^ | 1.000 | ,705^**^ | .276 | ,555^**^ | ,417^**^ | ,674^**^ | ,468^**^ | .083 | ,374^*^ | .132 | ,460^**^ | -.158 | .146 | .219 | .145 | ,390^**^ | .203 |  |
|  |  | Sig. (2-tailed) | .000 | .000 | .000 |  | .000 | .059 | .000 | .003 | .000 | .001 | .581 | .011 | .373 | .001 | .281 | .314 | .145 | .329 | .006 | .137 |  |
|  |  | N | 40 | 40 | 40 | 40 | 40 | 40 | 40 | 40 | 40 | 40 | 40 | 40 | 40 | 40 | 40 | 40 | 40 | 40 | 40 | 40 |  |
|  | Item 5 | Correlation Coefficient | ,465^**^ | ,490^**^ | ,620^**^ | ,705^**^ | 1.000 | ,388^**^ | ,383^**^ | .269 | ,605^**^ | ,711^**^ | .003 | ,454^**^ | .084 | ,453^**^ | -.120 | .222 | .144 | .237 | ,345^*^ | .110 |  |
|  |  | Sig. (2-tailed) | .001 | .000 | .000 | .000 |  | .008 | .006 | .061 | .000 | .000 | .983 | .002 | .575 | .002 | .417 | .126 | .340 | .114 | .016 | .422 |  |
|  |  | N | 40 | 40 | 40 | 40 | 40 | 40 | 40 | 40 | 40 | 40 | 40 | 40 | 40 | 40 | 40 | 40 | 40 | 40 | 40 | 40 |  |
|  | Item 6 | Correlation Coefficient | ,444^**^ | .258 | .269 | .276 | ,388^**^ | 1.000 | .238 | .033 | .183 | .239 | .225 | .264 | -.112 | .212 | .215 | ,382^*^ | .052 | .146 | .269 | -.082 |  |
|  |  | Sig. (2-tailed) | .002 | .073 | .062 | .059 | .008 |  | .099 | .826 | .223 | .112 | .149 | .082 | .466 | .150 | .157 | .011 | .739 | .343 | .068 | .562 |  |
|  |  | N | 40 | 40 | 40 | 40 | 40 | 40 | 40 | 40 | 40 | 40 | 40 | 40 | 40 | 40 | 40 | 40 | 40 | 40 | 40 | 40 |  |
|  | Item 7 | Correlation Coefficient | ,434^**^ | ,718^**^ | ,505^**^ | ,555^**^ | ,383^**^ | .238 | 1.000 | ,622^**^ | ,469^**^ | ,355^*^ | .113 | .215 | .019 | ,378^**^ | .184 | ,282^*^ | .039 | .012 | .102 | .182 |  |
|  |  | Sig. (2-tailed) | .002 | .000 | .000 | .000 | .006 | .099 |  | .000 | .001 | .013 | .448 | .136 | .897 | .007 | .204 | .049 | .793 | .935 | .468 | .177 |  |
|  |  | N | 40 | 40 | 40 | 40 | 40 | 40 | 40 | 40 | 40 | 40 | 40 | 40 | 40 | 40 | 40 | 40 | 40 | 40 | 40 | 40 |  |
|  | Item 8 | Correlation Coefficient | .276 | ,544^**^ | ,470^**^ | ,417^**^ | .269 | .033 | ,622^**^ | 1.000 | ,679^**^ | ,408^**^ | .110 | .036 | -.072 | .206 | -.064 | .090 | 0.000 | -.222 | .054 | .034 |  |
|  |  | Sig. (2-tailed) | .053 | .000 | .001 | .003 | .061 | .826 | .000 |  | .000 | .006 | .468 | .806 | .629 | .153 | .664 | .539 | 1.000 | .141 | .709 | .805 |  |
|  |  | N | 40 | 40 | 40 | 40 | 40 | 40 | 40 | 40 | 40 | 40 | 40 | 40 | 40 | 40 | 40 | 40 | 40 | 40 | 40 | 40 |  |
|  | Item 9 | Correlation Coefficient | ,390^**^ | ,443^**^ | ,513^**^ | ,674^**^ | ,605^**^ | .183 | ,469^**^ | ,679^**^ | 1.000 | ,635^**^ | .234 | .275 | -.117 | ,347^*^ | -.035 | .010 | .058 | -.186 | .206 | .056 |  |
|  |  | Sig. (2-tailed) | .007 | .002 | .000 | .000 | .000 | .223 | .001 | .000 |  | .000 | .130 | .067 | .443 | .017 | .815 | .947 | .707 | .225 | .160 | .690 |  |
|  |  | N | 40 | 40 | 40 | 40 | 40 | 40 | 40 | 40 | 40 | 40 | 40 | 40 | 40 | 40 | 40 | 40 | 40 | 40 | 40 | 40 |  |
|  | Item 10 | Correlation Coefficient | .259 | ,403^**^ | ,406^**^ | ,468^**^ | ,711^**^ | .239 | ,355^*^ | ,408^**^ | ,635^**^ | 1.000 | .216 | ,364^*^ | .097 | ,466^**^ | -.019 | .201 | -.177 | .104 | ,305^*^ | .028 |  |
|  |  | Sig. (2-tailed) | .074 | .005 | .005 | .001 | .000 | .112 | .013 | .006 | .000 |  | .163 | .016 | .526 | .001 | .897 | .178 | .254 | .497 | .037 | .845 |  |
|  |  | N | 40 | 40 | 40 | 40 | 40 | 40 | 40 | 40 | 40 | 40 | 40 | 40 | 40 | 40 | 40 | 40 | 40 | 40 | 40 | 40 |  |
|  | Item 11 | Correlation Coefficient | .080 | -.012 | -.006 | .083 | .003 | .225 | .113 | .110 | .234 | .216 | 1.000 | .288 | -.156 | .086 | .292 | .153 | -.096 | -.119 | .088 | .181 |  |
|  |  | Sig. (2-tailed) | .596 | .936 | .967 | .581 | .983 | .149 | .448 | .468 | .130 | .163 |  | .065 | .322 | .569 | .062 | .321 | .547 | .455 | .564 | .215 |  |
|  |  | N | 40 | 40 | 40 | 40 | 40 | 40 | 40 | 40 | 40 | 40 | 40 | 40 | 40 | 40 | 40 | 40 | 40 | 40 | 40 | 40 |  |
|  | Item 12 | Correlation Coefficient | .247 | .129 | ,353^*^ | ,374^*^ | ,454^**^ | .264 | .215 | .036 | .275 | ,364^*^ | .288 | 1.000 | .243 | ,389^**^ | .021 | ,388^**^ | -.111 | ,353^*^ | .108 | .211 |  |
|  |  | Sig. (2-tailed) | .092 | .372 | .014 | .011 | .002 | .082 | .136 | .806 | .067 | .016 | .065 |  | .114 | .008 | .890 | .010 | .476 | .023 | .465 | .138 |  |
|  |  | N | 40 | 40 | 40 | 40 | 40 | 40 | 40 | 40 | 40 | 40 | 40 | 40 | 40 | 40 | 40 | 40 | 40 | 40 | 40 | 40 |  |
|  | Item 13 | Correlation Coefficient | -.120 | -.076 | .171 | .132 | .084 | -.112 | .019 | -.072 | -.117 | .097 | -.156 | .243 | 1.000 | ,314^*^ | -.047 | .076 | -.085 | ,489^**^ | -.013 | .067 |  |
|  |  | Sig. (2-tailed) | .416 | .603 | .240 | .373 | .575 | .466 | .897 | .629 | .443 | .526 | .322 | .114 |  | .036 | .758 | .618 | .589 | .002 | .929 | .641 |  |
|  |  | N | 40 | 40 | 40 | 40 | 40 | 40 | 40 | 40 | 40 | 40 | 40 | 40 | 40 | 40 | 40 | 40 | 40 | 40 | 40 | 40 |  |
|  | Item 14 | Correlation Coefficient | ,320^*^ | ,333^*^ | ,354^*^ | ,460^**^ | ,453^**^ | .212 | ,378^**^ | .206 | ,347^*^ | ,466^**^ | .086 | ,389^**^ | ,314^*^ | 1.000 | .275 | .222 | -.076 | .265 | .222 | .151 |  |
|  |  | Sig. (2-tailed) | .024 | .018 | .011 | .001 | .002 | .150 | .007 | .153 | .017 | .001 | .569 | .008 | .036 |  | .063 | .128 | .617 | .078 | .122 | .272 |  |
|  |  | N | 40 | 40 | 40 | 40 | 40 | 40 | 40 | 40 | 40 | 40 | 40 | 40 | 40 | 40 | 40 | 40 | 40 | 40 | 40 | 40 |  |
|  | Item 15 | Correlation Coefficient | .008 | -.057 | -.227 | -.158 | -.120 | .215 | .184 | -.064 | -.035 | -.019 | .292 | .021 | -.047 | .275 | 1.000 | .238 | -.119 | .054 | -.269 | .064 |  |
|  |  | Sig. (2-tailed) | .959 | .695 | .116 | .281 | .417 | .157 | .204 | .664 | .815 | .897 | .062 | .890 | .758 | .063 |  | .112 | .444 | .725 | .069 | .652 |  |
|  |  | N | 40 | 40 | 40 | 40 | 40 | 40 | 40 | 40 | 40 | 40 | 40 | 40 | 40 | 40 | 40 | 40 | 40 | 40 | 40 | 40 |  |
|  | Item 16 | Correlation Coefficient | ,338^*^ | .246 | ,393^**^ | .146 | .222 | ,382^*^ | ,282^*^ | .090 | .010 | .201 | .153 | ,388^**^ | .076 | .222 | .238 | 1.000 | 0.000 | ,411^**^ | .007 | ,290^*^ |  |
|  |  | Sig. (2-tailed) | .020 | .085 | .006 | .314 | .126 | .011 | .049 | .539 | .947 | .178 | .321 | .010 | .618 | .128 | .112 |  | 1.000 | .007 | .965 | .039 |  |
|  |  | N | 40 | 40 | 40 | 40 | 40 | 40 | 40 | 40 | 40 | 40 | 40 | 40 | 40 | 40 | 40 | 40 | 40 | 40 | 40 | 40 |  |
|  | Item 17 | Correlation Coefficient | .101 | .059 | .150 | .219 | .144 | .052 | .039 | 0.000 | .058 | -.177 | -.096 | -.111 | -.085 | -.076 | -.119 | 0.000 | 1.000 | -.065 | -.051 | .066 |  |
|  |  | Sig. (2-tailed) | .504 | .693 | .312 | .145 | .340 | .739 | .793 | 1.000 | .707 | .254 | .547 | .476 | .589 | .617 | .444 | 1.000 |  | .684 | .736 | .652 |  |
|  |  | N | 40 | 40 | 40 | 40 | 40 | 40 | 40 | 40 | 40 | 40 | 40 | 40 | 40 | 40 | 40 | 40 | 40 | 40 | 40 | 40 |  |
|  | Item 18 | Correlation Coefficient | .124 | .064 | .229 | .145 | .237 | .146 | .012 | -.222 | -.186 | .104 | -.119 | ,353^*^ | ,489^**^ | .265 | .054 | ,411^**^ | -.065 | 1.000 | .004 | .227 |  |
|  |  | Sig. (2-tailed) | .407 | .663 | .118 | .329 | .114 | .343 | .935 | .141 | .225 | .497 | .455 | .023 | .002 | .078 | .725 | .007 | .684 |  | .978 | .116 |  |
|  |  | N | 40 | 40 | 40 | 40 | 40 | 40 | 40 | 40 | 40 | 40 | 40 | 40 | 40 | 40 | 40 | 40 | 40 | 40 | 40 | 40 |  |
|  | Item 19 | Correlation Coefficient | ,346^*^ | .245 | ,278^*^ | ,390^**^ | ,345^*^ | .269 | .102 | .054 | .206 | ,305^*^ | .088 | .108 | -.013 | .222 | -.269 | .007 | -.051 | .004 | 1.000 | .077 |  |
|  |  | Sig. (2-tailed) | .015 | .081 | .048 | .006 | .016 | .068 | .468 | .709 | .160 | .037 | .564 | .465 | .929 | .122 | .069 | .965 | .736 | .978 |  | .578 |  |
|  |  | N | 40 | 40 | 40 | 40 | 40 | 40 | 40 | 40 | 40 | 40 | 40 | 40 | 40 | 40 | 40 | 40 | 40 | 40 | 40 | 40 |  |
|  | Item 20 | Correlation Coefficient | ,282^*^ | .169 | ,267^*^ | .203 | .110 | -.082 | .182 | .034 | .056 | .028 | .181 | .211 | .067 | .151 | .064 | ,290^*^ | .066 | .227 | .077 | 1.000 |  |
|  |  | Sig. (2-tailed) | .039 | .210 | .047 | .137 | .422 | .562 | .177 | .805 | .690 | .845 | .215 | .138 | .641 | .272 | .652 | .039 | .652 | .116 | .578 |  |  |
|  |  | N | 40 | 40 | 40 | 40 | 40 | 40 | 40 | 40 | 40 | 40 | 40 | 40 | 40 | 40 | 40 | 40 | 40 | 40 | 40 | 40 |  |
| **. Correlation is significant at the 0.01 level (2-tailed). | | | | | | | | | | | | | | | | | | | | | | | |
| *. Correlation is significant at the 0.05 level (2-tailed). | | | | | | | | | | | | | | | | | | | | | | | |

**APPENDIX S17**

**ICC between Day 1 and Day 8 by Domain and Total AQUAREL**

| **ITEM** | **Intraclass Correlation** | **95% CI** | **Significance** |
| --- | --- | --- | --- |
|  | **Average Measure**  **(~Cronbach-α)** |  |  |
| CHS | 0.698 | 0.218 - 0.882 | 0.007 |
| DYS | 0.776 | 0.436 - 0.911 | 0.001 |
| ARR | 0.859 | 0.639 - 0.944 | 0.000 |
| TOTAL AQUAREL | 0.779 | 0.434 - 0.913 | 0.001 |

**APPENDIX S18**

***AQUAREL Questionnaire***

| 1. Have you felt discomfort in the chest?   - no discomfort at all - very mild discomfort - mild discomfort - moderate discomfort - great discomfort | 11. Have you been out of breath at rest?   - not out of breath - slightly out of breath - moderately out of breath - very out of breath - extremely out of breath |
| --- | --- |
| 2. Do you get chest discomfort while walking upstairs or uphill?   - no discomfort - very mild discomfort - mild discomfort - moderate discomfort - severe discomfort | 12. Do you awake when sleeping due to shortness of breath?   - never - seldom - once in awhile - often - continuously |
| 3. Do you get chest discomfort while walking quickly on level ground?   - no discomfort - very mild discomfort - mild discomfort - moderate discomfort - severe discomfort | 13. Did you have swollen ankles?   - never - seldom - once in awhile - often - continuously |
| 4. Do you get chest discomfort while walking on level ground at the same pace as people usually do at your age?   - no discomfort - very mild discomfort - mild discomfort - moderate discomfort - severe discomfort | 14. Have you suffered from an irregular heartbeat?   - never - seldom - once in awhile - often - continuously |
| 5. Have you been restricted by chest discomfort during physical exercise?   - Not restricted at all - Slightly restricted - Moderately restricted - Very restricted - Extremely restricted | 15. Have you suffered from heart pounding?   - never - seldom - once in awhile - often - continuously |
| 6. Have you experienced chest discomfort at rest?   - no discomfort - very mild discomfort - mild discomfort - moderate discomfort - severe discomfort | 16. Have you suffered from pounding in the neck or abdomen?   - never - seldom - once in awhile - often - continuously |
| 7. Do you get short of breath while walking upstairs or uphill?   - not short of breath - very mildly short of breath - mild short of breath - moderate short of breath - extreme short of breath | 17. Have you felt close to fainting?   - never - seldom - once in awhile - often - continuously |
| 8. Do you get short of breath while walking quickly on level ground?   - not short of breath - very mildly short of breath - mild short of breath - moderate short of breath - extreme short of breath | 18. Do you feel tired and exhausted after a night’s sleep?   - never - seldom - once in awhile - often - continuously |
| 9. Do you get short of breath while walking on level ground at the same pace as people usually do at your age?   - not short of breath - very mildly short of breath - mild short of breath - moderate short of breath - extreme short of breath | 19. Have you been restricted in your daily activities due to tiredness or lack of energy?   - extremely restricted - very restricted - moderately restricted - slightly restricted - not restricted at all |
| 10.Have you been restricted by breathlessness during physical exercise?   - not restricted at all - slightly restricted - moderately restricted - very restricted - extremely restricted | 20. Did you have to sit or lie down during the day to rest?   - never - seldom - once in awhile - often - continuously |

**APPENDIX S19**

**Initial English-Indonesia AQUAREL Translation 1**

| 1. Apakah Anda pernah merasakan rasa tidak nyaman pada dada?   - tidak ada rasa tidak nyaman sama sekali - rasa tidak nyaman sangat ringan - rasa tidak nyaman ringan - rasa tidak nyaman sedang - sangat merasa tidak nyaman | 11. Apakah Anda selama ini merasa sesak napas saat sedang istirahat?   - tidak sesak napas - sedikit sesak napas - cukup sesak napas - sangat sesak napas - amat sangat sesak napas |
| --- | --- |
| 2. Apakah Anda merasakan rasa tidak nyaman pada dada saat berjalan naik tangga atau menanjak?   - tidak ada rasa tidak nyaman - rasa tidak nyaman sangat ringan - rasa tidak nyaman ringan - rasa tidak nyaman sedang - sangat merasa tidak nyaman | 12. Apakah Anda terbangun saat tidur akibat sesak?   - tidak pernah - jarang - kadang-kadang - sering - selalu |
| 3. Apakah Anda merasakan rasa tidak nyaman pada dada saat berjalan cepat di tanah yang datar?   - tidak ada rasa tidak nyaman - rasa tidak nyaman sangat ringan - rasa tidak nyaman ringan - rasa tidak nyaman sedang - sangat merasa tidak nyaman | 13. Apakah Anda sebelumnya mengalami bengkak pada pergelangan kaki?   - tidak pernah - jarang - kadang-kadang - sering - selalu |
| 4. Apakah Anda merasakan rasa tidak nyaman pada dada saat berjalan di tanah yang datar dengan kecepatan yang sama seperti yang biasa dilakukan oleh orang lain seusia Anda?   - tidak ada rasa tidak nyaman - rasa tidak nyaman sangat ringan - rasa tidak nyaman ringan - rasa tidak nyaman sedang - sangat merasa tidak nyaman | 14. Apakah Anda pernah mengalami detak jantung yang tidak teratur?   - tidak pernah - jarang - kadang-kadang - sering - selalu |
| 5. Apakah Anda selama ini terbatas oleh rasa tidak nyaman pada dada selama olahraga?   - tidak terbatas sama sekali - sedikit terbatas - cukup terbatas - sangat terbatas - amat sangat terbatas | 15. Apakah Anda pernah mengalami jantung berdebar?   - tidak pernah - jarang - kadang-kadang - sering - selalu |
| 6. Apakah Anda pernah mengalami rasa tidak nyaman pada dada saat sedang istirahat?   - tidak ada rasa tidak nyaman - rasa tidak nyaman sangat ringan - rasa tidak nyaman ringan - rasa tidak nyaman sedang - sangat merasa tidak nyaman | 16. Apakah Anda pernah mengalami leher atau perut terasa berdenyut?   - tidak pernah - jarang - kadang-kadang - sering - selalu |
| 7. Apakah Anda merasa sesak napas saat berjalan naik tangga atau menanjak?   - tidak sesak napas - sesak napas sangat ringan - sesak napas ringan - sesak napas sedang - sesak napas berat | 17. Apakah Anda pernah merasa hampir pingsan?   - tidak pernah - jarang - kadang-kadang - sering - selalu |
| 8. Apakah Anda merasa sesak saat berjalan cepat di tanah yang datar?   - tidak sesak napas - sesak napas sangat ringan - sesak napas ringan - sesak napas sedang - sesak napas berat | 18. Apakah Anda merasa lelah dan kecapaian setelah tidur malam hari?   - tidak pernah - jarang - kadang-kadang - sering - selalu |
| 9. Apakah Anda merasa sesak saat berjalan di tanah yang datar dengan kecepatan yang sama seperti yang biasa dilakukan oleh orang lain seusia Anda?   - tidak sesak napas - sesak napas sangat ringan - sesak napas ringan - sesak napas sedang - sesak napas berat | 19. Apakah Anda selama ini merasa terbatas dalam melakukan aktivitas sehari-hari karena kelelahan atau kurang energi?   - amat sangat terbatas - sangat terbatas - cukup terbatas - sedikit terbatas - tidak terbatas sama sekali |
| 10.Apakah Anda selama ini merasa terbatas oleh sesak napas selama olahraga?   - tidak terbatas sama sekali - sedikit terbatas - cukup terbatas - sangat terbatas - amat sangat terbatas | 20. Apakah pada siang hari Anda harus duduk atau berbaring untuk beristirahat?   - tidak pernah - jarang - kadang-kadang - sering - selalu |

**APPENDIX S20**

**Initial English-Indonesia AQUAREL Translation 2**

| 1. Apakah Anda merasa dada Anda sesak?  • tidak ada rasa sesak sama sekali  • rasa sesak sangat ringan  • rasa sesak ringan  • rasa sesak sedang  • rasa sesak yang sangat hebat  2. Apakah Anda merasa dada Anda sesak ketika naik tangga atau berjalan pada jalanan menanjak?  • tidak ada rasa sesak  • rasa sesak sangat ringan  • rasa sesak ringan  • rasa sesak sedang  • rasa sesak yang sangat hebat  3. Apakah Anda merasa dada Anda sesak ketika berjalan cepat di jalan yang datar?  • tidak ada rasa sesak  • rasa sesak sangat ringan  • rasa sesak ringan  • rasa sesak sedang  • rasa sesak yang sangat hebat  4. Apakah Anda merasa dada Anda sesak ketika berjalan cepat di jalan yang datar dengan kecepatan yang sama seperti orang-orang pada usia Anda?  • tidak ada rasa sesak  • rasa sesak sangat ringan  • rasa sesak ringan  • rasa sesak sedang  • rasa sesak yang sangat hebat  5. Apakah Anda terkendala oleh rasa sesak di dada pada saat melakukan latihan fisik?  • sama sekali tidak terkendala  • sedikit terkendala  • cukup terkendala  • sangat terkendala  • sangat terkendala sekali  6. Apakah Anda pernah terkendala oleh rasa sesak di dada pada saat diam?  • tidak sama sekali  • rasa sesak sangat ringan  • rasa sesak ringan  • rasa sesak sedang   • rasa sesak yang sangat hebat  7. Apakah Anda terengah-engah ketika naik tangga atau berjalan pada jalanan menanjak?  • tidak terengah-engah  • terengah-engah sangat ringan  • terengah-engah ringan  • terengah-engah sedang  • sangat terengah-engah  8. Apakah Anda terengah-engah ketika berjalan cepat di jalan yang datar?  • tidak terengah-engah  • terengah-engah sangat ringan  • terengah-engah ringan  • terengah-engah sedang  • sangat terengah-engah  9. Apakah Anda terengah-engah ketika berjalan cepat di jalan yang datar dengan kecepatan yang sama seperti orang-orang pada usia Anda?  • tidak terengah-engah  • terengah-engah sangat ringan  • terengah-engah ringan  • terengah-engah sedang  • sangat terengah-engah  10. Apakah Anda pernah terkendala oleh kesulitan bernapas pada saat melakukan latihan fisik?  • sama sekali tidak terkendala  • sedikit terkendala  • cukup terkendala  • sangat terkendala  • sangat terkendala sekali | 11. Apakah Anda pernah terengah-engah pada saat diam?  • tidak terengah-engah  • sedikit terengah-engah  • terengah-engah sedang  • sangat terengah-engah  • terengah-engah secara luar biasa  12. Apakah Anda terbangun ketika tidur disebabkan oleh kehabisan napas/perasaan tertekan di dada?  • tidak pernah  • jarang  • kadang-kadang  • sering  • terus menerus  13. Apakah Anda mengalami pembengkakan pada pergelangan kaki?  • tidak pernah  • jarang  • kadang-kadang  • sering  • terus menerus  14. Apakah Anda pernah mengalami detak jantung yang tidak teratur?  • tidak pernah  • jarang  • kadang-kadang  • sering  • terus menerus  15. Apakah Anda pernah mengalami jantung yang berdebar keras?  • tidak pernah  • jarang  • kadang-kadang  • sering  • terus menerus  16 Apakah Anda pernah mengalami jantung yang berdebar keras yang terasa di leher atau perut?  • tidak pernah  • jarang  • kadang-kadang  • sering  • terus menerus  17. Apakah Anda pernah merasa hampir pingsan?  • tidak pernah  • jarang  • kadang-kadang  • sering  • terus menerus  18. Apakah Anda merasa lelah dan kehabisan tenaga setelah tidur malam?  • tidak pernah  • jarang  • kadang-kadang  • sering  • terus menerus  19. Apakah Anda pernah terkendala dalam kegiatan sehari-hari Anda karena kelelahan atau kekurangan tenaga?  • sangat terkendala sekali  • sangat terkendala  • cukup terkendala  • sedikit terkendala  • tidak terkendala sama sekali  20. Apakah Anda harus duduk atau berbaring pada siang hari untuk beristirahat?  • tidak pernah  • jarang  • kadang-kadang  • sering  • terus menerus |
| --- | --- |

**APPENDIX S21**

**Translation Synthesis AQUAREL Questionnaire**

1. Apakah Anda merasakan rasa tidak nyaman pada dada?

- tidak ada rasa tidak nyaman sama sekali
- rasa tidak nyaman sangat ringan
- rasa tidak nyaman ringan
- rasa tidak nyaman sedang
- rasa tidak nyaman sangat hebat

1. Apakah Anda merasakan rasa tidak nyaman pada dada saat naik tangga atau berjalan menanjak?

- tidak ada rasa tidak nyaman
- rasa tidak nyaman sangat ringan
- rasa tidak nyaman ringan
- rasa tidak nyaman sedang
- rasa tidak nyaman sangat hebat

1. Apakah Anda merasakan rasa tidak nyaman pada dada saat berjalan cepat di jalan yang datar?

- tidak ada rasa tidak nyaman
- rasa tidak nyaman sangat ringan
- rasa tidak nyaman ringan
- rasa tidak nyaman sedang
- rasa tidak nyaman sangat hebat

1. Apakah Anda merasakan rasa tidak nyaman pada dada saatberjalan di jalan yang datar dengan kecepatan yang sama seperti orang lain seusia Anda?

- tidak ada rasa tidak nyaman
- rasa tidak nyaman sangat ringan
- rasa tidak nyaman ringan
- rasa tidak nyaman sedang
- rasa tidak nyaman sangat hebat

1. Apakah Anda selama ini terkendala oleh rasa tidak nyaman di dada selama melakukan latihan fisik?

- sama sekali tidak terkendala
- sedikit terkendala
- cukup terkendala
- sangat terkendala
- amat sangat terkendala

1. Apakah Anda pernah mengalami rasa tidak nyaman di dada saat sedang istirahat?

- tidak ada rasa tidak nyaman
- rasa tidak nyaman sangat ringan
- rasa tidak nyaman ringan
- rasa tidak nyaman sedang
- rasa tidak nyaman sangat hebat

1. Apakah Anda sesak napas saat naik tangga atau berjalan menanjak?

- tidak sesak napas
- sesak napas sangat ringan
- sesak napas ringan
- sesak napas sedang
- sesak napas sangat berat

1. Apakah Anda sesak napas saat berjalan cepat di jalan yang datar?

- tidak sesak napas
- sesak napas sangat ringan
- sesak napas ringan
- sesak napas sedang
- sesak napas sangat berat

1. Apakah Anda sesak napas saat berjalan di jalan yang datar dengan kecepatan yang sama seperti orang lain seusia Anda?

- tidak sesak napas
- sesak napas sangat ringan
- sesak napas ringan
- sesak napas sedang
- sesak napas sangat berat

1. Apakah Anda selama ini terkendala oleh sesak napas selama melakukan latihan fisik?

- sama sekali tidak terkendala
- sedikit terkendala
- cukup terkendala
- sangat terkendala
- amat sangat terkendala

1. Apakah Anda selama ini sesak napas saat sedang istirahat?

- tidak sesak napas
- sesak napas sangat ringan
- sesak napas ringan
- sesak napas sedang
- sesak napas sangat berat

1. Apakah Anda terbangun saat sedang tidur karena sesak napas?

- tidak pernah
- jarang
- kadang-kadang
- sering
- terus-menerus

1. Apakah Anda sebelumnya mengalami bengkak pada pergelangan kaki?

- tidak pernah
- jarang
- kadang-kadang
- sering
- terus menerus

1. Apakah Anda pernah mengalami detak jantung yang tidak teratur?

- tidak pernah
- jarang
- kadang-kadang
- sering
- terus menerus

1. Apakah Anda pernah mengalami jantung yang berdebar keras?

- tidak pernah
- jarang
- kadang-kadang
- sering
- terus menerus

1. Apakah Anda pernah mengalami leher atau perut terasa berdenyut?

- tidak pernah
- jarang
- kadang-kadang
- sering
- terus menerus

1. Apakah Anda pernah merasa hampir pingsan?

- tidak pernah
- jarang
- kadang-kadang
- sering
- terus menerus

1. Apakah Anda merasa lelah dan kehabisan tenaga setelah tidur malam?

- tidak pernah
- jarang
- kadang-kadang
- sering
- terus menerus

1. Apakah Anda selama ini merasa terkendala dalam melakukan kegiatan sehari-hari karena kelelahan atau kurang tenaga?

- amat sangat terkendala
- sangat terkendala
- cukup terkendala
- sedikit terkendala
- tidak terkendala sama sekali

20. Apakah Anda harus duduk atau berbaring pada siang hari untuk beristirahat?

- tidak pernah
- jarang
- kadang-kadang
- sering
- terus menerus

**APPENDIX S22**

**Back Translation AQUAREL Questioannaire 1**

1. Do you feel any discomfort in your chest?

- I have no discomfort at all
- I have a very light feeling of discomfort
- I have a light feeling of discomfort
- I have a moderate feeling of discomfort
- I have a very great feeling of discomfort

1. Do you feel any discomfort in your chest when climbing stairs or walking uphill?

- I have no discomfort
- I have a very light feeling of discomfort
- I have a light feeling of discomfort
- I have a moderate feeling of discomfort
- I have a very great feeling of discomfort

1. Do you feel any discomfort in your chest when walking quickly on a flat road?

- I have no discomfort
- I have a very light feeling of discomfort
- I have a light feeling of discomfort
- I have a moderate feeling of discomfort
- I have a very great feeling of discomfort

1. Do you feel any discomfort in your chest when walking on a flat road at the same speed as other people of your age?

- I have no discomfort
- I have a very light feeling of discomfort
- I have a light feeling of discomfort
- I have a moderate feeling of discomfort
- I have a very great feeling of discomfort

1. Have you ever felt constrained by discomfort in your chest when doing physical exercise?

- I have never felt constrained at all
- I have felt a bit constrained
- I have felt quite constrained
- I have felt very constrained
- I have felt extremely constrained

1. Have you ever felt any discomfort in your chest while resting?

- I have never felt discomfort
- I have had a very light feeling of discomfort
- I have had a light feeling of discomfort
- I have had a moderate feeling of discomfort
- I have had a very great feeling of discomfort

1. Do you get short of breath when climbing stairs or walking uphill?

- I don’t get short of breath
- I get very slightly short of breath
- I get slightly short of breath
- I get moderately short of breath
- I get very short of breath

1. Do you get short of breath when walking quickly on a flat road?

- I don’t get short of breath
- I get very slightly short of breath
- I get slightly short of breath
- I get moderately short of breath
- I get very short of breath

1. Do you get short of breath when walking on a flat road at the same speed as other people of your age?

- I don’t get short of breath
- I get very slightly short of breath
- I get slightly short of breath
- I get moderately short of breath
- I get very short of breath

1. Have you ever felt constrained by shortness of breath when doing physical exercise?

- I have never felt constrained at all
- I have felt a bit constrained
- I have felt quite constrained
- I have felt very constrained
- I have felt extremely constrained

1. Have you ever felt shortness of breath while resting?

- I’ve never felt short of breath
- I’ve felt very slightly short of breath
- I’ve felt slightly short of breath
- I’ve felt moderately short of breath
- I’ve felt very short of breath

1. Have you ever woken up from sleep due to a shortness of breath?

- Never
- Rarely
- Sometimes
- Often
- Continually

1. Have you ever experienced swelling in your ankles before?

- Never
- Rarely
- Sometimes
- Often
- Continually

1. Have you ever experienced an irregular heartbeat?

- Never
- Rarely
- Sometimes
- Often
- Continually

1. Have you ever experienced a pounding heart?

- Never
- Rarely
- Sometimes
- Often
- Continually

1. Have you ever experienced pulsations in your neck or stomach?

- Never
- Rarely
- Sometimes
- Often
- Continually

1. Have you ever felt you were about to faint?

- Never
- Rarely
- Sometimes
- Often
- Continually

1. Do you still feel tired and lethargic after waking up in the morning?

- Never
- Rarely
- Sometimes
- Often
- Continually

1. Have you ever felt constrained in carrying out your daily activities because of tiredness or a lack of energy?

- I’ve felt extremely constrained
- I’ve felt very constrained
- I’ve felt quite constrained
- I’ve felt a bit constrained
- I’ve never felt constrained at all

20. Do you have to sit or lie down during the day to take a rest?

- Never
- Rarely
- Sometimes
- Often
- Continually

**APPENDIX S23**

**Back Translation AQUAREL Questioannaire 2**

1. Do you feel discomfort in your chest?

- no discomfort
- very slight discomfort
- slight discomfort
- moderate discomfort
- extreme discomfort

1. Do you feel discomfort in your chest when climbing stairs or walking uphill?

- no discomfort
- very slight discomfort
- slight discomfort
- moderate discomfort
- extreme discomfort

1. Do you feel discomfort in your chestwhen walking fast on a flat surface?

- no discomfort
- very slight discomfort
- slight discomfort
- moderate discomfort
- extreme discomfort

1. Do you feel discomfort in your chestwhen walking on a flat surface at the same speed as other people your age?

- no discomfort
- very slight discomfort
- slight discomfort
- moderate discomfort
- extreme discomfort

1. Do you ever feel constrained by discomfort in your chest when doing physical exercise?

- Not at all
- Slightly
- Somewhat
- Very much
- Extremely

1. Do you ever experience discomfort in your chest when resting?

- no discomfort
- very slight discomfort
- slight discomfort
- moderate discomfort
- extreme discomfort

1. Do you feel short of breath when climbing stairs or walking uphill?

- Not at all
- very slightly short of breath
- slightly short of breath
- moderately short of breath
- extremely short of breath

1. Do you feel short of breath when walking fast on a flat surface?

- Not at all
- very slightly short of breath
- slightly short of breath
- moderately short of breath
- extremely short of breath

1. Do you feel short of breath when walking on a flat surface at the same speed as other people your age?

- Not at all
- very slightly short of breath
- slightly short of breath
- moderately short of breath
- extremely short of breath

1. Do you ever feel constrained by shortness of breath when doing physical exercise?

- Not at all
- Slightly constrained
- Somewhat constrained
- very constrained
- extremely constrained

1. Do you ever feel short of breath when resting?

- Not at all
- very slightly short of breath
- slightly short of breath
- moderately short of breath
- extremely short of breath

1. Are you ever awakened from sleep by shortness of breath?

- never
- seldom
- occasionally
- often
- constantly

1. Did you previously experience swelling in your ankles?

- never
- seldom
- occasionally
- often
- constantly

1. Do you ever experience irregular heartbeat?

- never
- seldom
- occasionally
- often
- constantly

1. Do you ever experience strongly pounding heartbeat?

- never
- seldom
- occasionally
- often
- constantly

1. Do you ever feel throbbing in your neck or stomach?

- never
- seldom
- occasionally
- often
- constantly

1. Do you ever feel as if you are going to pass out?

- never
- seldom
- occasionally
- often
- constantly

1. Do you ever feel exhausted and out of energy before going to bed at night?

- never
- seldom
- occasionally
- often
- constantly

1. Do you ever feel constrained in doing your daily activities by fatigue or lack of energy?

- Extremely constrained
- Very constrained
- Somewhat constrained
- Slightly constrained
- Not at all

20. Do you have to sit or lie down to rest during the day?

- never
- seldom
- occasionally
- often
- constantly
